# Intravenous ketamine for subacute treatment of refractory chronic migraine: a case series

**DOI:** 10.1186/s10194-016-0700-3

**Published:** 2016-11-22

**Authors:** Clinton Lauritsen, Santiago Mazuera, Richard B. Lipton, Sait Ashina

**Affiliations:** 1 Department of Neurology, Thomas Jefferson University, Jefferson Headache Center, Philadelphia, PA USA; 2 Department of Neurology, Montefiore Headache Center, Albert Einstein College of Medicine, Bronx, NY USA; 3 Department of Neurology, New York University School of Medicine, NYU Langone Medical Center, NYU Lutheran Headache Center, New York, NY USA

## Abstract

**Background:**

Refractory migraine is a challenging condition with great impact on health related quality of life. Intravenous (IV) ketamine has been previously used to treat various refractory pain conditions. We present a series of patients with refractory migraine treated with intravenous ketamine in the hospital setting.

**Methods:**

Based on retrospective chart review, we identified six patients with refractory migraine admitted from 2010 through 2014 for treatment with intravenous ketamine. Ketamine was administered using a standard protocol starting with a dose of 0.1 mg/kg/hr and increased by 0.1 mg/kg/hr every 3 to 4 h as tolerated until the target pain score of 3/10 was achieved and maintained for at least 8 h. Visual Analogue Scale (VAS) scores at time of hospital admission were obtained as well as average baseline VAS scores prior to ketamine infusion. A phone interview was conducted for follow-up of migraine response in the 3 to 6 months following ketamine infusion.

**Results:**

The study sample had a median age of 36.5 years (range 29–54) and 83% were women. Pre-treatment pain scores ranged from 9 to 10. All patients achieved a target pain level of 3 or less for 8 h; the average ketamine infusion rate at target was 0.34 mg/kg/hour (range 0.12–0.42 mg/kg/hr). One patient reported a transient out-of-body hallucination following an increase in the infusion rate, which resolved after decreasing the rate. There were no other significant side effects.

**Conclusion:**

IV ketamine was safely administered in the hospital setting to patients with refractory chronic migraine. Treatment was associated with short term improvement in pain severity in 6 of 6 patients with refractory chronic migraine. Prospective placebo-controlled trials are needed to assess short term and long-term efficacy of IV ketamine in refractory chronic migraine.

**Electronic supplementary material:**

The online version of this article (doi:10.1186/s10194-016-0700-3) contains supplementary material, which is available to authorized users.

## Background

Chronic migraine and refractory migraine have long challenged clinicians. In the United States, chronic migraine prevalence is nearly 1% and results in enormous impact on headache-related disability, including higher Migraine Disability Assessment Test (MIDAS), reduced health-related quality of life (HRQoL), increased depression and anxiety (PHQ-4 and GAD-7 respectively), compared to episodic migraine [1–6]. Chronic migraine has also been shown to result in greater economic burden and health care resource utilization [1, 3, 7–9]. Annually, the total economic cost of chronic migraine is over three time that of episodic migraine, when consider direct medical costs and loss from decreased productivity [8].

Refractory migraine is included in ICHD-2 [10] and ICHD-3 beta [11] but criteria for intractable headache have only recently emerged [12]. Silberstein et al. [12] assesses the type and number of treatments the patient failed as well as the clinical setting and the intensity of invention for intractable headache. Multiple treatment options have been proposed for management of refractory migraine including intravenous dihydroergotamine (DHE) and intravenous divalproex sodium [13, 14].

One candidate treatment for intractable migraine is ketamine. Intravenous ketamine has been studied in various refractory pain conditions including complex regional pain [15]. Intranasal ketamine reduced the severity of aura in migraine with prolonged aura in a small randomized trial [16]. The use of intravenous ketamine has only been reported in case series. Krusz et al. [17] demonstrated improvement in pain scores in patients with refractory migraine as well as few side effects associated with treatment.

Ketamine is a dissociative anesthetic that acts on glutamate binding sites at the N-methyl-D-aspartate (NMDA) receptor as well as at opioid, monoaminergic, cholinergic, nicotinic, and muscarinic receptors [18]. There is a theory of functional and electrophysiological dissociation between thalamo-neocortical and limbic systems: sensory inputs may reach cortical receiving areas, but fail to be observed in some of the association areas with the use of ketamine [18]. Analysis of the dose-dependent ketamine effects on pain processing showed a decreasing activation of the secondary somatosensory cortex, insula, and anterior cingulate cortex, which has been linked to the affective pain component that underlines the potency of ketamine in modulating affective pain processing [19]. This theoretical mechanism of action of ketamine has shown to decrease central sensitization and allodynia in pain conditions, which has motived clinicians to use it as treatment for migraine. Ketamine also reduces cortical spreading depression in animal models [20]. Most common known side effects of ketamine may include cardiovascular instability, respiratory changes and psychiatric symptoms including acute psychosis, hallucinations, anxiety but are usually dose-dependent.

Herein, we report the effect of IV ketamine on a series of six patients with intractable migraine treated with escalated doses on an inpatient basis.

## Methods

We performed a retrospective chart review study. From 2010 until 2014, a total of six patients were admitted for treatment with continuous intravenous ketamine to Mount Sinai Beth Israel Hospital in New York. Data were collected from charts obtained from the medical record department and electronic medical records (PRISM). The study was approved by the Mount Sinai Institutional Review Board. Prior to admission, a diagnosis of chronic migraine without aura based on ICHD-2 criteria was made by a headache specialist (Sait Ashina). Demographics, number of prior migraine treatments and current use of medications such as opioids, antidepressants, beta blockers, antiepileptic medications and nonsteroidal anti-inflammatory drugs (NSAIDs) were documented, as well as onset age of migraine diagnosis.

All the patients received information regarding side effects and risks associated with the treatment and informed written consent was obtained prior to infusion. Monitoring of the treatment was done according to the ketamine infusion procedure policy of the Department of Pain Medicine and Palliative Care at Mount Sinai Beth Israel (see Additional file 1 for sources used in protocol development). Following an initial electrocardiogram (EKG) for all patients and a pregnancy test for female patients, ketamine was administered using a standard protocol starting with an initial intravenous infusion of 0.1 mg/kg/hr. The infusion was increased by 0.1 mg/kg/hr every 3 to 4 h as tolerated until the goal pain score of 3/10 was maintained for 8 h. The eight-hour designation was made based on standard pain assessment intervals in the institution. Thereafter, the infusion was decreased by 0.2 mg/kg/hr every 3to 4 h until the infusion rate reached 0 mg/kg/hr. The dose of ketamine escalated as needed until maximal response or until undesirable side effects including psychomimetic and dysphoric effects.

Visual Analogue scale (VAS) score was used at the moment of admission and follow-up VAS scores at different ketamine infusion rates were assessed from nursing and infusion records. We defined a pain response as a reduction in the initial VAS to a score of three or less. Side effects were identified and reported.

We attempted to contact the patients in this retrospective study for telephone follow-up; however, we were only able to reach two of the six patients. During telephone interview, a questionnaire including MIDAS [21] was administered.

## Results

Characteristics of patients admitted for ketamine are summarized in Table 1. Ages ranged from 29 to 54 (median of 36.5) and five out of six were women. VAS scores on admission were nine or ten (see Table 1). All of the patients were Caucasian. The median age of migraine onset was 17 (range 8–29). The median duration of illness was 17 years (range 12–46). The mean number of failed acute migraine treatments was 18 (range 14–26) and failed preventive medications was 25 (range 7–29) (See Table 2). Three patients out of the total six receiving ketamine had a prior psychiatric diagnosis (depression, panic attacks and/or borderline personality disorders). In addition to chronic migraine, concomitant chronic pain disorders were identified in three of six patients, two of whom also had a psychiatric diagnosis. The six patients were on at least one of the following medications: muscle relaxant, NSAID, opioid, antiepileptic, antidepressant, benzodiazepine, triptan, beta blocker or antiemetic (Table 2).

**Table 1:** The demographic and clinical data and intravenous (IV) ketamine infusion rates at which 6 patients achieved a VAS pain score of 3 or less

Patient	Age/sex	Age at migraine onset	Duration of Illness	Psychiatric and Pain Comorbidities	Initial VAS	IV Ketamine Rate VAS < 3	Time to maintain VAS of 3 or less for duration of 8 h
1	42/F	26	16 years	Depression, Panic Attacks, Chronic Back Pain	10	0.42 mg/kg/hr	36 h
2	29/F	15	14 years	Panic Attacks	10	0.38 mg/kg/hr	40 h
3	31/M	19	12 years	Complex Regional Pain Syndrome	9	0.41 mg/kg/hr	20 h
4	54/F	8	46 years	None	9	0.12 mg/kg/hr	12 h
5	30/F	10	20 years	Depression, Borderline Personality Disorder, Chronic Neck Pain	9	0.35 mg/kg/hr	73 h
6	47/F	29	18 years	Depression	9	0.34 mg/kg/hr	82 h

**Table 2:** Outpatient medical regimen in patients with chronic migraine cases at time of scheduled treatment with intravenous ketamine

Patient	1	2	3	4	5	6
Opioid	1		1		1	
Non-steroidal anti-inflammatory	1		2			1
Anti-depressant		1		2	1	2
Muscle relaxant		1			1	1
Benzodiazepine			1	1		
Anti-emetic		1	1		1	1
Neuroleptic					2	1
Anti-epileptic		2				1
Triptan						1
Anti-hypertensive				1	1	1

All of the six patients were started on IV ketamine dose of 0.1 mg/kg/hr. All six patients achieved the target endpoint of a pain score of less than three out of ten sustained for at least 8 h. The intravenous ketamine doses are presented in Table 1. We provide the current outpatient migraine regimens at the time of admission as well as the number of acute and preventive migraine treatments failed for each patient in Tables 2 and 3. Telephone follow-up was obtained in just two patients, neither of whom reported sustained benefits from intravenous ketamine infusion. One patient reported an out of body hallucination which resolved following decrease in the infusion rate.

**Table 3:** Number of previously failed medications for each patient

Patient	1	2	3	4	5	6
Abortive Medications Failed	19	NA	16	14	18	26
Preventive Medications Failed	25	5	17	28	23	29
OnabotulinumtoxinA Failed	Yes	NA	Yes	Yes	Yes	Yes

*NA* not available

## Discussion

In this small cases series, all six patients with refractory migraine met the target pain relief endpoint with ketamine over a mean infusion of 44 h (range 12–82). Mean ketamine infusion rate at the time of pain relief endpoint was 0.34 mg/kg/hour (range 0.12–0.42). Patients achieved pain relief without substantial adverse effects. One patient reported a brief dissociative experience, which reversed.

Intravenous ketamine use in treating refractory depression has recently been well established [22, 23]. Studies have also suggested a role for ketamine in the treatment of intractable chronic pain including long-term analgesic effect persisting beyond the duration of infusion [24]. Long-term ketamine infusion (4–14 days) has been shown to decrease pain for up to 3 months [25]. Allodynia, a marker of chronic pain and central sensitization of nociceptive pathways, has also been shown decrease with intravenous ketamine infusion. Interestingly, this effect was not achieved until 4–5 days of continuous infusion [26].

The existing literature on IV ketamine for the treatment of headache is limited. Though our case series is modest in size, and in the absence of a contemporaneous placebo group, causal inferences are not possible, we demonstrated short-term success in pain relief in intractable migraine patients with one significant but short-lived adverse event. It is biologically plausible that ketamine could be an effective treatment for intractable headache. Ketamine is an antagonist at NMDA receptors, blocking the excitatory action of glutamate (Glu), a neurotransmitter long implicated in the pathophysiology of migraine [27]. Glu has been shown to be implicated in induction of cortical spreading depression (CSD), activation of trigeminal nociceptive neurons as well as play a role in central sensitization. Previous studies have identified variants in the gene for GluA receptors in persons with migraine [28].

To date, there has been a lack of treatments with reliable abortive effect on migraine aura, the phenomenon attributed to CSD. In the rat-model, both ketamine and the non-specific NMDA antagonist, MK-801 have been shown to block CSD, demonstrated electrophysiologically and by fMRI [29]. In a double-blinded, randomized parallel-group controlled study of 18 patients with migraine with aura, Afridi et al. tested the effect of intranasal ketamine compared to midazolam on aura. Ketamine was shown to reduce severity of aura but not duration, whereas midazolam had no effect [16]. Intranasal ketamine has been shown to consistently improve aura symptoms in some patients with familial hemiplegic migraine, although without significant reduction of headache severity [30]. Note: Broadly blocking CSD with long term administration is viewed as a model for preventive treatment [31]. Memantine is a voltage-dependent noncompetitive antagonist at the glutamatergic NMDA receptor, which inhibits the prolonged influx of calcium associated with neuronal excitotoxicity. In order to identify an agent with preventive activity against refractory and chronic migraine, Bigal, et al. administered daily memantine to 28 patients in an unblinded protocol. Compared to baseline, at 3 months, memantine decreased headache frequency severe headaches and MIDAS scores [32]. Ketamine may be a particularly beneficial treatment option for patients that have failed memantine. Because ketamine is the most potent competitive antagonist at the NMDA-type glutamate receptor whereas memantine is a weaker and noncompetitive antagonist, ketamine may have greater impact on central sensitization [33]. Also of interest, in patients concomitantly treated with opioids, ketamine has been shown to increase pain relief.[34] This may suggest a role for ketamine in the treatment of medication overuse headache.

Of the patients that completed follow up questionnaires, none reported lasting benefit from ketamine 3–6 months post infusion. Prior publications have reported lasting effects on chronic non-headache pain reduction following long term infusions of more than 4 days. Of note, none of the patients included in this case series received ketamine infusion for more than 4 days. Thus, we propose that future studies target ketamine infusions for at least this duration. Once placebo controlled studies on acute headache relief are performed, studies assessing long-term benefits should begin. Strategies for maintaining the effect of intravenous ketamine infusion should also be studied, such as the ongoing administration of a daily or as needed NMDA receptor antagonist. Researchers have used similar strategies in prior studies, such as with the use of mexiletine following lidocaine infusion for chronic daily headache. In this fashion, agents including dextromethorphan-quinidine, memantine, oral or intranasal ketamine could be used to maintain the benefit from NDMA receptor antagonism following ketamine infusion [35, 36].

## Co﻿nclusions

﻿This study highlights the need for further research regarding new treatment options for patients who suffer daily consequences of refractory migraine and have failed many abortive and preventive medications. Our IV ketamine infusion protocol, based on gradually dose escalation, relieves pain without substantial adverse effects. However, future study of this benefit on short-term headache relief needs to be conducted in a placebo-controlled fashion and this publication may serve as the basis for the design of such a trial.

## Additional file

Additional file 1: Sources used for the development of the Mount Sinai Beth Israel Hospital protocol for IV ketamine infusion. (DOC 23 kb)
