# Supplementary material for: Intravenous ketamine for subacute treatment of refractory chronic migraine: a case series
Source: J Headache Pain. 2016 Nov 22;17(1):106. doi: 10.1186/s10194-016-0700-3 (PMC5120050; doi:10.1186/s10194-016-0700-3)
Supplement: Additional file 1: — Sources used for the development of the Mount Sinai Beth Israel Hospital protocol for IV ketamine infusion. (DOC 23 kb) [file 10194_2016_700_MOESM1_ESM.doc]

**Additional file 1:** Sources used for the development of the Mount Sinai Beth Israel Hospital protocol for IV ketamine infusion

1. Campbell-Fleming, J. and Williams, A. (2008). The use of ketamine as adjuvant therapy to control severe pain. Clinical Journal of Oncology Nursing, 12(1), 102-108.
2. Correll, G., Maleki, J., Hestermann, M., Gracely, E., Muir, J., & Harbut, R. (2004).
3. Subanesthetic ketamine infusion therapy: A retrospective analysis of a novel therapeutic approach to complex regional pain syndrome. Pain medicine, 5(3), 263-275.
4. Hocking, G. and Cousins, M. (2003). Ketamine in chronic pain management: An evidence based review. Anesthesia and Analgesia, (97), 1730-9.
5. McCaffrey, M. and Passero, C. (1999). Pain: Clinical Manual (2nd ed., pp. 336-338). St. Louis, MO: Mosby
